# Effects of TNF receptor blockade on in vitro cell survival and response to negative energy balance in dairy cattle

**DOI:** 10.1186/s40104-017-0224-y

**Published:** 2018-01-10

**Authors:** C. A. Martel, L. K. Mamedova, J. E. Minton, M. Garcia, C. Legallet, B. J. Bradford

**Affiliations:** Department of Animal Sciences and Industry, Kansas State University, 135 Call Hall, Manhattan, KS 66506 USA

**Keywords:** Dairy cows, Fatty liver, Glucose tolerance test, Tumor necrosis factor α

## Abstract

**Background:**

Associative data and some controlled studies suggest that the inflammatory cytokine tumor necrosis factor (TNF) α can induce fatty liver in dairy cattle. However, research demonstrating that TNFα is a necessary component in the etiology of bovine fatty liver is lacking. The aim of this work was to evaluate whether blocking TNFα signaling with a synthetic cyclic peptide (TNF receptor loop peptide; TRLP) would improve liver metabolic function and reduce triglyceride accumulation during feed restriction.

**Results:**

Capability of TRLP to inhibit TNFα signaling was confirmed on primary bovine hepatocytes treated with recombinant bovine TNFα and 4 doses of TRLP (0, 1, 10, 50 μmol/L) over 24 h. Next, 4 lactating Holstein cows (parity 1.4 ± 0.5, 433 ± 131 d in milk) in an incomplete Latin rectangle design (3 × 2) were subcutaneously administered with different TRLP doses (0, 1.5, 3.0 mg/kg BW) every 4 h for 24 h, followed by an intravenous injection of TNFα (5 μg/kg BW). Before and for 2 h after TNFα injection, TRLP decreased plasma non-esterified fatty acid (NEFA) concentration (*P* ≤ 0.05), suggesting an altered metabolic response to inflammation. Finally, 10 non-pregnant, non-lactating Holstein cows (3.9 ± 1.1 yr of age) were randomly assigned to treatments: control (carrier: 57% DMSO in PBS) or TRLP (1.75 mg TRLP /kg BW per day). Treatments were administrated every 4 h for 7 d by subcutaneous injection to feed-restricted cows fed 30% of maintenance energy requirements. Daily blood samples were analyzed for glucose, insulin, β-hydroxybutyrate, NEFA, and haptoglobin concentrations, with no treatment effects detected. On d 7, cows completed a glucose tolerance test (GTT) by i.v. administration of a dextrose bolus (300 mg glucose/kg BW). Glucose, insulin, and NEFA responses failed to demonstrate any significant effect of treatment during the GTT. However, plasma and liver analyses were not indicative of dramatic lipolysis or hepatic lipidosis, suggesting that the feed restriction protocol failed to induce the metabolic state of interest. Injection site inflammation, assessed by a scorer blinded to treatment, was enhanced by TRLP compared to control.

**Conclusions:**

Although the TRLP inhibited bovine TNFα signaling and altered responses to i.v. administration of TNFα, repeated use over 7 d caused apparent local allergic responses and it failed to alter metabolism during a feed restriction-induced negative energy balance. Although responses to feed restriction seemed atypical in this study, side effects of TRLP argue against its future use as a tool for investigating the role of inflammation in metabolic impacts of negative energy balance.

## Background

The proinflammatory cytokine tumor necrosis factor alpha (TNFα) has been implicated in several metabolic disorders, including fatty liver disease and insulin resistance in dairy cattle [1, 2]. Our laboratory has conducted several studies to confirm that TNFα-induced inflammation alters metabolic function in dairy cows. For cows in late lactation, TNFα infusion promoted hepatic triglyceride (TG) accumulation, with changes in hepatic mRNA profiles consistent with a shift from lipid oxidation to storage [3]. In periparturient cows, TNFα increased circulating haptoglobin and reduced dry matter intake and milk yield, albeit without altering liver TG content [4]. Tumor necrosis factor alpha elicits its effects on tissues and organs after binding one of its 2 receptors (TNFR: TNFR1 or TNFR2). Activation of TNFR can lead to the activation of caspases [5], NF-κB [6], and MAP-kinase pathways [7], which have significant roles in altering metabolic pathways. If TNFα does not bind to its receptors, activation of inflammatory pathways involved in alteration of organ function does not take place. Receptor antagonists and TNFα blocking peptides like TNF receptor loop peptide (TRLP) work by interfering with TNFα activation of its corresponding TNFR [8]. The inability of TNFα to trigger signaling downstream of its receptor abrogates most of its effects.

Numerous studies have evaluated blocking TNFα activation and signaling in vivo, primarily via administration of antibodies against TNFα [9] and by genetic deletion of TNFR [10]. Other work has investigated how blocking TNFα affects bone resorption, rheumatoid arthritis [11], and neurodegenerative disorders [12], all diseases that associated with chronic inflammation and tissue damage. In one such study, Saito et al. [13] used the cyclic peptide TRLP to block the effects of TNFα in mice with collagen-induced arthritis. Administration of TRLP delayed the onset of arthritis and inhibited RANKL-induced signaling, which mediates TNFα-induced arthritic damage. Reducing circulating TNFα concentrations by genetic alteration [14] or immunodepletion [9] has beneficial effects on glucose metabolism in obesity, and similar strategies could benefit metabolic function and health in cows undergoing negative energy balance.

Although previous work has demonstrated that exogenous TNFα can promote changes in liver metabolism consistent with the development of fatty liver [3], that does not necessarily imply that endogenous TNFα is an essential factor in the natural development of this disorder. To test the essentiality of TNFα signaling for fatty liver development during negative energy balance in dairy cows, we used TRLP to block TNFα signaling during an induced negative energy balance. We hypothesized that subcutaneous injections of TRLP would limit TNFR1 signaling and partially prevent the development of insulin resistance and hepatic lipidosis during feed restriction in obese cows. The objectives were to 1) verify the ability of TRLP to block bovine TNFα/TNFR signaling, 2) determine the dose of TRLP required to alter in vivo responses to bovine TNFα, and 3) evaluate the effects of TRLP on lipolysis, liver TG accumulation, and glucose metabolism in energy-restricted dairy cows.

## Methods

### Experiment 1: In vitro validation of TRLP in bovine hepatocytes

The Kansas State University Biotechnology Core chemically synthesized TRLP [8]. The synthesized peptide (YCWSQYLCY) was agitated in a basic solution (pH 8.0) for 3 d to induce internal disulfide bonding, and the resulting cyclic peptide was purified by HPLC (>90% purity) and lyophilized. Mass spectrometry verified that TRLP had the appropriate mass.

To provide a source of cells expressing the bovine TNFR, bovine hepatocytes were isolated from a steer (~12 mo of age) at slaughter [15]. Within 15 min after euthanasia by captive bolt, approximately 25 g of liver tissue were excised and immediately placed in liver perfusion medium (Invitrogen Corp., Carlsbad, CA) supplemented with penicillin-streptomycin. Using a syringe and needle, the tissue was perfused with 500 mL of the medium until blanched, approximately 5 min. Following perfusion, tissue was minced. The minced tissue then digested with collagenase type 1 (Worthington-Biochemical Corp., Lakewood, NJ) at 37 °C for 90 min. The suspension was centrifuged at 50×*g* for 3 min to pellet cells. The cells pellet was then washed 3 times in 50 mL of hepatocyte wash medium (Invitrogen Corp.). Washed cells were resuspended in 10 mL of wash medium and counted using a hemacytometer. Viability was assessed by trypan blue exclusion. Cells were centrifuged one more time and the pellet resuspended in William’s medium E (Invitrogen Corp.) containing 5% fetal bovine serum (CELLect Gold; MP Biomedicals, Solon, OH) and plated (1.5 × 10^6^ cells/well) on 24-well culture plates (Corning Costar, Fisher Scientific, Waltham, MA).

Hepatocytes were allowed to attached for 24 h at 37 °C, then media and unattached cells were aspirated and media including cycloheximide (1 μg/mL; Sigma-Aldrich, St. Louis, MO) was added to all wells to promote apoptosis by TNFα [16]. Four wells (500 μL/well) were assigned to each of the following treatments: control, recombinant bovine TNFα (25 ng/mL), TRLP (100 μmol/L), or TNFα (25 ng/mL) + TRLP (1, 10, or 50 μmol/L) treatments. Cells were incubated with the appropriate treatments for 24 h before the relative proportion of dead cells was determined by propidium iodide staining [16].

### Experiment 2: In vivo dose determination

#### Animals and experimental design

Previous studies performed in rodents reported an effective dose of TRLP administered at 2 mg/kg BW [13, 17], and these findings were used as a starting point to evaluate doses in the cow. Four lactating Holstein cows (BW: 672 ± 153 kg, parity: 1.4 ± 0.5, days in milk: 433 ± 131) were enrolled in a 4 × 4 Latin square design balanced for carryover effects. Cows were housed in a tie-stall facility for 2 d before injections allowing for adaptation, where they were fed a lactation diet formulated to meet all nutritional requirements [18] and milked 3 times/d in a parlor. A 3-day resting time was allowed between periods. Cows were assigned to 1 of 4 TRLP doses (0, 0.75, 1.5, and 3 mg/kg BW per day). The daily dose was divided into 6 subcutaneous injections delivered every 4 h for 24 h. Solubility of TRLP in PBS was poor, so TRLP was solubilized with 11% dimethyl sulfoxide (DMSO; Sigma-Aldrich Co. St. Louis, MO) in PBS at a final concentration of 14.4 mg/mL. Varying doses were delivered by varying the volume of TRLP solution injected. After 24 h of TRLP administration, cows were challenged with recombinant bovine TNFα at 5 μg/kg BW as an intravenous bolus [19]. Recombinant bovine TNFα was custom-produced in an *E. coli* expression system (Genscript Corp., Piscataway, NJ) as previously described [3].

Although originally arranged to be a 4 × 4 Latin square, in period 1, the cow assigned to 0 mg TRLP /kg BW responded quite strongly to TNFα administration. Whereas the other 3 cows on the experiment had apparently recovered by 36 to 48 h after TNFα challenge, the fourth cow did not return to normal DMI even 5 d after the TNFα challenge. Others have reported that cattle typically recover from this protocol in less than 24 h [19]; therefore, we decided to replace that cow for the second period. In period 2, the replacement cow was also assigned to the same TRLP treatment (0 mg/kg of BW) to provide a basis for comparison in subsequent periods. This cow responded even more dramatically than the cow which had been removed in period 1. Approximately 40 h after treatment, the cow was observed seizing. Per veterinarian recommendation, the cow was treated with dexamethasone and i.v. fluids, but died shortly after. As in period 1, the remaining 3 cows recovered satisfactorily and were apparently normal within 30 h post-TNFα challenge. After this, the experiment was suspended with a distribution of 3, 1, 2, and 2 cows in treatments 0, 0.75, 1.5, and 3 mg/kg BW TRLP, respectively. Therefore, for subsequent statistical analysis the dose of 0.75 mg TRLP /kg BW as well as all data from the cow that died in period 2 were excluded from analysis, leaving a 3 × 2 Latin rectangle with 3 treatments and 2 observations for each treatment.

#### Sample analyses

Blood samples were collected from the coccygeal vein into evacuated tubes (Vacutainer, Becton Dickinson, Franklin Lakes, NJ, USA) containing potassium EDTA (TNFα and NEFA analyses) or potassium oxalate with sodium fluoride as a glycolytic inhibitor (glucose analysis). Blood samples were collected at 0 (approximately 2 h after morning feeding), 1, 2, and 3 h post-TNFα bolus, based on temporal responses reported previously [19]. Plasma was separated by centrifugation at 2000×*g* for 10 min immediately after collection and stored at −20 °C. Colorimetric kits were used to quantify glucose and NEFA in plasma samples as previously described [3]. Concentrations of TNFα were measured using a sandwich ELISA procedure as previously described [20].

### Experiment 3: Evaluating the role of TNFα in feed restriction-induced fatty liver

#### Animals and experimental design

Ten non-pregnant, non-lactating Holstein cows (BW: 685 ± 64 kg, age: 3.9 ± 1.1 yr) were randomly assigned to one of two treatments: control (57% DMSO in PBS) or TRLP (1.75 mg/kg BW TRLP daily, dissolved in 57% DMSO in PBS). A greater proportion of DMSO was used in the diluent for this experiment to allow for increased concentration of TRLP and smaller injection volumes; both control (DMSO/PBS carrier) and TRLP were delivered at 1.2 μL/kg BW per injection. Treatment doses were administrated by subcutaneous injections every 4 h for 7 d to maintain relatively stable TRLP concentrations throughout the day. Treatments were administrated along the neck, alternating between the left and right side, to help prevent injection site irritation. Cows were housed in a tie-stall facility for 7 d before injections began, allowing for adaption to the facility. During the adaptation period, cows were fed an alfalfa and prairie hay based diet to provide 16.0 Mcal/d NE_L_ (100% of estimated maintenance energy requirements; Table 1). One day before injection treatments began, cows were placed on a restricted diet providing 30% of estimated maintenance energy requirements, and this ration was fed for the 7-day injection period (Table 1). The feed restriction protocol was modeled after that of Cooke et al. [21], where it induced a 10-fold increase in liver TG content. Cows were fed twice per day (08:00 and 16:00 h) during both the adaptation and treatment periods, and water was offered ad libitum.

**Table 1:** Ingredient and nutrient composition and intake of adaptation and restricted diets fed to non-pregnant non-lactating Holstein cows in experiment 3

Item	Adaptation phase	Restriction phase
Ingredient composition, % of DM		
Corn silage	31.8	14.6
Alfalfa hay	31.6	17.1
Prairie hay	31.6	52.1
Grain mix^a^	4.95	15.1
Salt		1.01
Nutrient composition		
Dry matter, % as fed^b^	50.4	49.9
Crude protein, % DM	13.6	11.9
Neutral detergent fiber, % DM	45.9	49.1
Amount provided		
kg DM/d	10.7	3.46 ± 0.20
Mcal NE_L_/d	16.0	4.8 ± 0.3

^a^Grain mix contained 75% dry-rolled corn grain, 15% dicalcium phosphate, 4.5% sodium bicarbonate, 4.0% vitamin E premix (44 IU/g), 0.76% trace mineral salt, 0.42% vitamin A premix (30 kIU/g), 0.12% vitamin D premix (30 kIU/g), and 0.076% selenium premix (600 ppm)

^b^Water was added to both TMR diets to reduce the DM content to 50%

#### Data and sample collection

Blood samples were collected daily (13:00 h) during the 7-day injection period. Two blood samples were collected from the coccygeal vein into evacuated tubes containing potassium EDTA (for insulin, β-hydroxybutyrate [BHBA], NEFA, and haptoglobin analyses) or potassium oxalate (Vacutainer, Becton Dickinson) for analysis of glucose. On d 5, jugular catheters were surgically placed to prepare for a glucose tolerance test (GTT). On d 7, after the daily blood sample collection, cows were infused with a sterile solution of 50% dextrose (wt/vol) at a dose of 300 mg glucose/kg BW in less than 5 min via intra-jugular catheter [22]. Blood samples were collected from the jugular vein 10 min before infusion and at 10 min intervals through 120 min post-infusion. At the conclusion of the GTT, liver biopsies were collected for analysis of liver TG concentration. Sample collection and surgical methods were carried out as described by Morey et al. [23].

On d 5, before jugular catheter placement, a technician blinded to treatments scored injection sites on both sides of the neck for degree of tissue inflammation. A scoring system from 0 to 5 was used, with 0 indicating no visible signs of irritation or lumps, and 5 indicating serious irritation (4 + lumps). At the initiation of the treatments and at the conclusion of the study, all cows were weighed to determine weight loss during the restriction period.

#### Plasma analyses

Colorimetric kits were used to quantify glucose, NEFA, and BHBA concentrations in all plasma samples as previously described [3]. A bovine-specific ELISA was used to quantify insulin (Bovine Insulin ELISA; Mercodia AB Sweden). Plasma haptoglobin was measured by bovine-specific ELISA (Life Diagnostics, West Chester, PA, USA) with one modification during the dilution stage; instead of a 2000-fold dilution, a 3000-fold dilution was utilized. Glucose, NEFA, and insulin data from the GTT were used to determine the area under the curve by the trapezoidal rule for each variable, using the mean of the −10 and 0-min time points as a baseline value [22]. Additionally, insulin sensitivity was estimated by determining the Revised Quantitative Insulin Sensitivity Check Index value [24], quantified as RQUICKI = 1/[log(G_b_) + log(I_b_) + log(NEFA_b_)], where G_b_ is the blood plasma concentration of glucose in mg/dL, I_b_ is the blood plasma concentration of insulin in μU/mL, and NEFA_b_ is the blood plasma concentration of NEFA in mmol/L.

#### Liver analyses

Approximately 20 mg of liver was placed in 500 μL of chilled PBS (pH 7.4) and homogenized. The homogenate was centrifuged at 2000×*g* for 10 min at 4 °C and 100 μL of the supernatant was then removed for free glycerol and total protein analysis. Triglyceride content was measured using a method adapted from Starke et al. [25]. The remaining liver homogenate was incubated with 100 μL of lipase (porcine pancreatic lipase, MP Biomedicals, Santa Ana, CA) for 16 h at 37 °C, and glycerol content was then determined by an enzymatic glycerol phosphate oxidase method (Sigma-Aldrich Co.). Triglyceride content was calculated based on the difference between glycerol concentrations before and after lipase digestion. Total protein content of the original homogenate was analyzed by a Coomassie blue [26] colorimetric method (Thermo Scientific, Pierce, Rockford, IL). Liver TG concentration was normalized by protein concentration, which is unaltered in fatty liver [27], to avoid potential bias introduced by differences in water content of liver samples.

### Statistical analysis

#### Experiment 1

Cytotoxicity was analyzed as a completely randomized design with the MIXED procedure of SAS (SAS Institute, Cary, NC). The model included the fixed effect of treatment. The linear effect of increasing dose of TRLP (0, 1, 10, and 50 μmol/L) was evaluated using appropriate contrast coefficients for unequally spaced treatments, obtained using PROC IML in SAS. Significance was declared at *P* < 0.05.

#### Experiment 2

Due to suspension of the experiment after the death of a cow at the end of period 2, only data from a total of 6 cow periods were analyzed using PROC MIXED of SAS. The model included the fixed effects of treatment, time, and time × treatment interaction, and the random effects of cow and period. Results for NEFA and TNFα were log_10_-transformed to achieve normal residual distributions before statistical analysis and reported LSM and SEM were back transformed. Repeated measures over time were modeled with a heterogeneous autoregressive covariance structure, which was selected based on the Bayesian information criterion. Contrasts were used to test the effect of TRLP administration (across doses) and the effect of dose. Temporal responses to treatments were further examined using the contrasts at specific time points when the overall treatment or treatment × time *F*-tests were significant (*P* < 0.05).

#### Experiment 3

Data were analyzed as a completely randomized design using the PROC MIXED procedure of SAS with the fixed effects of treatment, day, and treatment by day interaction, and the random effect of cow. For all variables, data for the day of treatment initiation were used as covariate values. Daily measures of insulin, NEFA, haptoglobin, and RQUICKI were log_10_-transformed to achieve normal residual distributions before statistical analysis and reported LSM and SEM were back transformed. Repeated measures over time were modeled with a heterogeneous autoregressive covariance structure. Single time point measures were modeled with the fixed effect of treatment. Significance was declared at *P* < 0.05 and tendencies at *P* < 0.10.

## Results and discussion

### Experiment 1

**Fig. 1:**
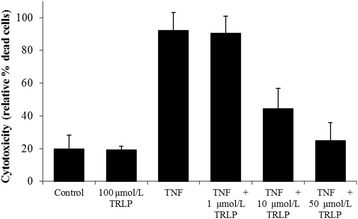
Experiment 1: Blockade of TNFα signaling by TNF receptor loop peptide (TRLP; linear dose effect: *P* < 0.01) in primary bovine hepatocytes. Hepatocytes were incubated with TNFα (25 ng/mL) and/or varying doses of TRLP for 24 h (*n* = 4). Cell death was measured by propidium iodide staining (means ± SD)

As expected, TNFα (25 ng/mL) induced (*P* < 0.01) the death of bovine primary hepatocytes whereas TRLP alone, even at 100 μmol/L, did not (*P* = 0.95, Fig. 1). In a dose-dependent manner, TRLP prevented TNFα-induced cytotoxicity (linear effect, *P* < 0.01), and at 50 μmol/L it completely blocked the effect of TNFα on cell death (no different than control, *P* = 0.51). These data demonstrate that TRLP blocks bovine TNFR interactions with potency equivalent to that observed in cultured human [8] and mouse [28] cells. Therefore, the potential ability of TRLP to block the downstream signaling of bovine TNFα in vivo merited further evaluation.

### Experiment 2

In vivo studies performed in rodents confirmed that TRLP has an inhibitory effect on TNFα-mediated inflammatory response [11, 13, 17], and we modeled our doses after these studies. However, as the metabolism and response to inflammatory/anti-inflammatory stimuli may vary by species, we performed a dose response experiment to identify the dose that significantly attenuates TNFα-mediated lipolysis, primarily by measuring circulating concentrations of NEFA.

**Fig. 2:**
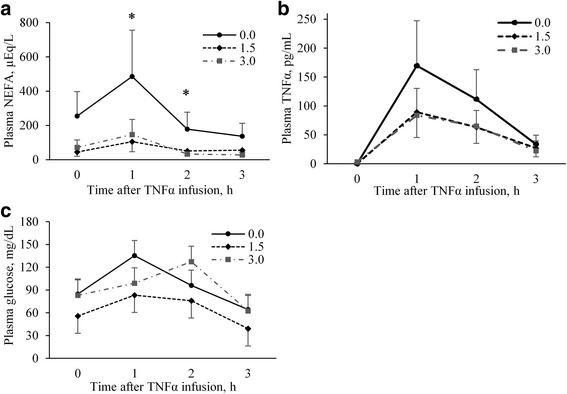
Experiment 2: Plasma concentrations of NEFA, TNFα, and glucose in cows (*n* = 2) infused with TNFα (5 μg/kg BW) after 24 h of administration of one of 3 doses (0, 1.5, 3 mg/kg BW per d) of TNF receptor loop peptide (TRLP). **a** TRLP effect: *P* < 0.001; dose effect: *P* = 0.66; time effect: *P* = 0.14; *TRLP effect at time point: *P* < 0.05. **b** TRLP effect: *P* = 0.81; dose effect: *P* = 0.41; time effect: *P* < 0.001. **c** TRLP effect: *P* = 0.49; dose effect: *P* = 0.14; time effect: *P* = 0.04. Interactions of treatment and time were not significant for these variables

Administration of TRLP reduced plasma NEFA concentration (overall effect *P* < 0.001), but dose had no effect (*P* = 0.66). There was no statistical difference in plasma NEFA between TRLP treatments and control prior to TNFα infusion (*P* = 0.12), but the post-TNFα lipolytic response was blunted by TRLP, with significant effects at 1 h and 2 h post-infusion and a tendency for an effect at 3 h (Fig. 2a). No dose effect was detected at any time point. Concentrations of plasma TNFα were very low before TNFα infusion (< 3.4 pg/mL, Fig. 2b), but at 1 h after infusion, plasma TNFα concentration peaked at 108 ± 30 pg/mL (time *P* < 0.001). No significant TRLP or dose effects were observed for plasma TNFα. Plasma glucose concentrations increased at 1 h after TNFα administration (*P* = 0.01; Fig. 2c) before decreasing, with concentrations at 3 h post-infusion significantly less than at baseline (*P* = 0.02). Plasma glucose concentration was not affected by TRLP or dose. Although we acknowledge the limited statistical power in this truncated experiment, we nevertheless observed effects of TRLP on plasma NEFA and failed to find any differences in response to 1.5 vs. 3.0 mg TRLP/kg BW, suggesting that 1.5 mg/kg BW was a sufficient dose for the subsequent experiment.

Determination of specific lipolytic signaling mechanisms affected by TRLP was not the focus of this study. However, TNFα stimulates lipolysis via signaling cascades mediated by PKA and MEK/ERK [29, 30], and it is logical that inhibition of TNFR1 signaling would prevent the activation of these second messengers. Recent studies [31, 32] concluded that TRLP exerts anabolic effects by regulating the RANK/RANKL signaling to induce bone formation rather than resorption. Furthermore, TNFα blockade with an anti-TNF antibody restored growth hormone signaling in murine colitis and improved anabolic metabolism [33].

### Experiment 3

Dramatic changes in nutrient demands and poor appetite during the transition to lactation can lead to dramatically increased plasma NEFA concentrations [34], leading to greater flux of FA into the liver. Coupled with saturable oxidative capacity and limited efficacy for TG export of the bovine liver [35, 36], this change in nutrient flux has traditionally been used to explain the common occurrence of fatty liver disease in early lactation dairy cows. It remains unclear whether inflammation contributes to the pathological manifestation of this phenomenon [37]. Ideally, TRLP would be evaluated in periparturient cows to test the impact of TNFα blockade in the animals of interest, but the great variance between animals during this time, and the increased sample size required as a result, makes this approach cost-prohibitive. We therefore turned to a feed restriction model [21, 38] to evaluate the impact of TRLP on mechanisms associated with the development of fatty liver.

**Fig. 3:**
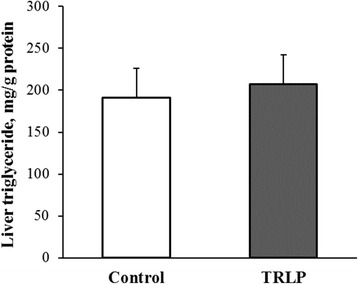
Experiment 3: Effect of subcutaneous injections of TNFα receptor loop peptide (TRLP, 1.75 mg/kg BW per day for 7 d) or carrier (Control) on liver triglyceride concentration in feed-restricted cows (*n* = 5). *P =* 0.76

Based on results from experiment 2, a dose of 1.75 mg TRLP /kg BW per day was used for 7 d in cows fed to meet 30% of maintenance energy requirements. Treatment did not alter hepatic TG content (191 vs. 207 ± 35.4 mg/g protein, for control and TRLP cows, respectively, *P* = 0.76, Fig. 3). Cooke et al. [21] observed a 10-fold increase in liver TG content after 10 d of feed restriction, but in the current experiment, liver TG concentrations were much less compared with previous studies in our laboratory, where mean TG concentrations were 770 and 900 mg/g protein in ad libitum-fed lactating cows [3, 4]. Feed restriction significantly increased plasma BHBA (235 vs. 162 ± 17 μmol/L, *P* < 0.001) and NEFA (509 vs. 337 ± 19 μmol/L, *P* < 0.001) concentrations over time, but treatment had no effects on plasma concentrations of glucose, insulin, BHBA, and NEFA (Table 2). Cows lost weight during the restriction week, but weight loss did not differ by treatment (58.8 vs. 45.2 ± 9.8 kg for CON vs. TRLP; *P* = 0.36).

**Table 2:** Effects of subcutaneous injections of TNFα receptor loop peptide (TRLP)^a^ on plasma metabolites in energy restricted cows^b^ (*n* = 5)

Item^c^	Treatment			*P*-value	
	Control	TRLP	SEM	TRT	TRT × day
Glucose, mg/dL	59.0	58.5	0.3	0.28	0.98
Insulin^d^, ng/mL	0.42	0.58	0.11	0.14	0.85
BHBA, μmol/L	235	218	16	0.48	0.19
NEFA, μEq/L	409	443	27	0.34	1.00
Haptoglobin^d^, μg/mL	27.6	54.9	12.5	0.27	0.31
RQUICKI^e^	0.55	0.50	0.03	0.04	0.98

^a^Cows were administered subcutaneous injections of TRLP at 1.75 mg/kg BW per day or carrier (Control) every 4 h for 7 d

^b^Cows were fed a diet restricted to provide 40% of estimated energy requirements for maintenance

^c^Values at enrollment were used as covariate

^d^Log_10_ transformation was used for statistical analysis and back-transformed LS means and SEM are presented

^e^Revised Quantitative Insulin Sensitivity Check Index, calculated as: 1/ [log(glucose, mg/dL) + log(insulin, μU/L) + log(NEFA, mmol/L)] (Holtenius and Holtenius, 2007)

The acute phase protein haptoglobin is released by the liver in response to inflammatory mediators [39] and was found to be increased in cows with fatty liver [40, 41]. Plasma haptoglobin concentrations, however, were not altered over time or by treatment (*P* = 0.27, Table 2). The observed values were less than concentrations reported for cows undergoing inflammation, such as those in the first week postpartum [4].

TNFα is known to induce insulin resistance in cattle [1, 2]. Furthermore, a recent review [42] concluded that the therapeutic use of TNFα inhibitors improves insulin sensitivity in patients with rheumatoid arthritis. Thus, if TNFα concentrations are naturally elevated during energy restriction, we hypothesized that blocking its effects would enhance insulin sensitivity. However, calculated RQUICKI values were decreased in TRLP-treated cows, indicative of reduced insulin sensitivity [24]. The validity of the RQUICKI metric remains questionable, though, and so we also used the GTT, which generates findings that correlate well with hyperinsulinemic euglycemic clamp results [43]. Results of the GTT revealed no treatment effects on glucose, insulin, or NEFA dynamics (Table 3).

**Table 3:** Effects of subcutaneous injections of TNFα receptor loop peptide (TRLP)^a^ on glucose tolerance test (GTT) responses in energy restricted cows^b^ (*n* = 5)

	Infusion Treatment			*P*- value
	Control	TRLP	SEM	
Glucose response, mg/dL × min				
AUC^c^	4024	4223	462.8	0.77
AUC^d^	5226	4932	738.5	0.79
Insulin response, ng/mL × min				
AUC^c^	77.3	73.9	32.6	0.94
AUC^d^	151.8	140.9	44.3	0.86
NEFA response, μEq/L × min				
AUC^c^	−4092	−2103	1081	0.25
AUC^d^	−14,674	−11,427	2034	0.31

^a^Cows were administered daily subcutaneous injections of TRLP at 1.75 mg/kg BW/d distributed in 6 doses daily (every 4 h) for 7 d

^b^Cows were fed a diet restricted to provide 30% of estimated energy requirements for maintenance

^c^Incremental area under the curve at 60 min post-dextrose bolus

^d^Incremental area under the curve at 120 min post-dextrose bolus

Overall, we found little evidence of an impact of TRLP on carbohydrate and lipid metabolism during the feed restriction period. However, the low hepatic TG concentrations and minimal changes in plasma NEFA and BHBA concentrations observed in cows in this experiment, regardless of treatment, suggest that the metabolic stress induced by our feed restriction protocol was minimal. Stable plasma haptoglobin concentrations during the restriction period likewise failed to reveal an hepatic inflammatory response, despite evidence that feed restriction alone can trigger such responses in lactating cows [44].

Major drawbacks of synthetic peptides are their low solubility and short half-life [45]. Due to the poor solubility of TRLP and to facilitate its diffusion to systemic circulation after subcutaneous injection [46], we used a high concentration of DMSO (57% solution) as carrier and control. Administration of DMSO is claimed to have anti-inflammatory effects, and it is important to consider whether administration of this compound to all animals provided sufficient anti-inflammatory effects to mitigate metabolic and inflammatory stress typically associated with feed restriction. However, across 6 injections, the total DMSO dose was only 4.5 mg/kg BW daily, which is approximately 1% of the dose evaluated previously for potential systemic anti-inflammatory effects [47].

A recent review points out that injection site reactions are the most frequent adverse side effect of anti-TNF therapeutics administered via subcutaneous infusions [48]. It would not be surprising if the foreign compound TRLP generated a local inflammatory response. Indeed, visual assessment of injection sites by a blinded evaluator demonstrated that TRLP caused a greater degree of injection-site irritation compared with control on d 5 (inflammation score: 2.9 vs. 0.1 ± 0.35, *P* < 0.01). Therefore, the potential systemic impact of TNFα blockage by TRLP must be considered in light of its apparent pro-inflammatory effect, at least at the local level. We did not find reports of injection-site reactions in previous papers using TRLP in vivo. It is possible that such reactions occurred and were simply not reported. However, it is also possible that the administration frequency in this study enhanced the allergic response to TRLP, or that administration of a dose appropriate for a mature dairy cow resulted in a local concentration at the injection site that was great enough to cause a negative response that was not triggered in mice given far lesser doses. Regardless, the injection site inflammation complicates interpretation of responses to TRLP, and any future work with this compound in cattle should be designed to minimize the risk of this effect as much as possible.

## Conclusions

Dose response experiments demonstrated that TRLP can block the cytotoxic effect of bovine TNFα in vitro and can attenuate the lipolytic effect of TNFα in cows. The intentions of the third experiment were to trigger the development of fatty liver in nonlactating cows through feed restriction, while using TRLP to determine whether blocking TNFα signaling would prevent this pathology. However, despite inducing an increase in lipolysis and BW loss, feed restriction did not promote meaningful accumulation of liver lipids or an inflammatory response. Coupled with observations of a local inflammatory response to TRLP, the failure of the fatty liver induction protocol limits our ability to reach conclusions regarding the effects of TNFα blockade during negative energy balance in dairy cattle. Therefore, questions remain regarding the necessity of TNFα signaling in the etiology of bovine fatty liver disease.

## Abbreviations

AUC: Area under the curve
BHBA: β-hydroxybutyric acid
DMI: Dry matter intake
DMSO: Dimethyl sulfoxide
EDTA: Ethylenediaminetetraacetic acid
ELISA: Enzyme-linked immunosorbent assay
FBS: Fetal bovine serum
GTT: Glucose tolerance test
HPLC: High-pressure liquid chromatography
LSM: Least-squares mean
NEFA: Non-esterified fatty acid
PBS: Phosphate-buffered saline
RQUICKI: Revised quantitative insulin sensitivity check index
SEM: Standard error of the mean
TG: Triglyceride
TNF: Tumor necrosis factor
TNFR: Tumor necrosis factor receptor
TRLP: Tumor necrosis factor receptor loop peptide
